# Comorbidity and Functional Trajectories From Midlife to Old Age: The Health and Retirement Study

**DOI:** 10.1093/gerona/glu113

**Published:** 2014-07-24

**Authors:** Sari Stenholm, Hugo Westerlund, Jenny Head, Martin Hyde, Ichiro Kawachi, Jaana Pentti, Mika Kivimäki, Jussi Vahtera

**Affiliations:** ^1^Department of Public Health, University of Turku, Turku, Finland.; ^2^Department of Health, Functional Capacity and Welfare, National Institute for Health and Welfare (THL), Helsinki, Finland.; ^3^Stress Research Institute, Stockholm University, Stockholm, Sweden.; ^4^Department of Epidemiology and Public Health, University College London, London.; ^5^Department of Society, Human Development, and Health, Harvard School of Public Health, Boston, Massachusetts.; ^6^Centre of Expertise for the Development of Work and Organizations, Finnish Institute of Occupational Health, Helsinki and Turku, Finland.; ^7^Turku University Hospital, Turku, Finland.

**Keywords:** Aging, Comorbidity, Physical functioning, Disability.

## Abstract

**Background.:**

The number of diseases and physical functioning difficulties tend to increase with age. The aim of this study was to examine the trajectories of physical functioning across age groups and whether the trajectories differ according to disease status in different population subgroups.

**Methods.:**

Repeat data from a nationally representative population sample, the Health and Retirement Study, was used. Participants were 10,709 men and 13,477 women aged 60–107 years at baseline with biennial surveys from 1992 to 2010. Average length of follow-up was 10.3 years ranging from 0 to 18 years. Disease status and physical functioning was asked about at all study phases and 10 items were summed to obtain a physical functioning score (0–10).

**Results.:**

Age modified the relationship between number of chronic diseases and physical functioning with older participants having more physical functioning difficulties with increasing number of diseases. An average 70-year-old participant with no diseases had 0.89 (95% CI: 0.85–0.93) physical functioning difficulties, with one disease 1.72 (95% CI: 1.69–1.76) difficulties, with two diseases 2.57 (95% CI: 2.52–2.62) difficulties, and with three or more diseases 3.82 (95% CI: 3.76–3.88) difficulties. Of the individual diseases memory-related diseases, stroke, pulmonary diseases, and arthritis were associated with significantly higher physical functioning difficulties compared with other diseases.

**Conclusions.:**

Comorbidity is associated with greater burden of physical functioning difficulties. Of the studied diseases, memory-related diseases, stroke, pulmonary diseases, and arthritis alone or in combination limit most physical functioning.

Physical functioning is an essential aspect of everyday life and enables autonomy and participation in meaningful social, cultural, and physical activities. Loss of physical functioning seriously threatens the independence and quality of life of older people (1). Persons with functional limitations are at increased risk of falls, institutionalization, and premature mortality (1–4). Preventing declines in physical functioning is of fundamental importance for both individuals and populations. Clinical and epidemiological studies conducted over the past three decades have identified multiple risk factors for functional decline including different diseases, physiological and psychological functions, health behaviors, and sociodemographic factors (5–7).

There is a robust evidence that several chronic diseases, including heart disease, stroke, chronic pulmonary disease, diabetes, cancer, osteoarthritis, depression, and cognitive impairment can lead to difficulties in physical functioning (8–10). Moreover, as the number of concurrent diseases increases, there is an increasing risk of functional limitations and disability (8,11–15). Some studies also suggest that interaction between certain diseases may predispose to greater functional decline than a combination of some other diseases (16).

Most of the previous research compares the relative risk of functional limitations among those with or without diseases and has been based on cross-sectional studies or longitudinal studies with only two time points (13). The observed associations are not always linear and therefore it is important to understand how functional limitations from midlife to old age develop over time. It is also of interest to examine how these developmental trajectories vary with emerging chronic conditions and in various population subgroups.

The Health and Retirement Study (HRS) is a longitudinal cohort study of retirement and health among a representative sample of older people in the United States. The extraordinarily rich and complex data with repeated measurements of exposure and outcome variables provides an opportunity to examine the trajectories of physical functioning across age groups and whether the trajectories differ according to disease status. We also examined the role of sociodemographic characteristics in the association between morbidity and physical functioning.

## Methods

### Participants

The HRS is an ongoing cohort study of Americans, with interview data collected biennially on demographics, health behavior, health status, employment, income and wealth, and insurance status. The first cohort was interviewed in 1992 and every 2 years subsequently, with five additional cohorts added in the phases in between 1994 and 2010. The full details of the study are described elsewhere (17). Ethical approval for the HRS Study was obtained from the University of Michigan Institutional Review Board.

In this study, we used data from 1992 to 2010 including participants aged 60–107 years, *n* = 24,186 (10,709 men and 13,477 women) and a total of 127,061 person-years of observation. The average length of follow-up was 10.2 years, ranging from 0 to 18 years.

### Measurement of Chronic Diseases

Eight time-varying chronic diseases, previously found to be associated with physical functioning (6,8,9), were included in our analyses. At each study wave, the participants were asked: “Has a doctor ever told you that you have...?” (1) heart disease (myocardial infarction, coronary heart disease, angina, congestive heart failure, or other heart problems) (2), stroke (3), chronic pulmonary disease (chronic bronchitis or emphysema) (4), cancer (a malignant tumor of any kind except skin cancer) (5), diabetes (diabetes or high blood sugar) (6), arthritis (arthritis or rheumatism) (7), and memory-related disease. Information on the first six diseases was available in each study wave (1–10), but memory-related diseases were inquired only in Waves 4–9.

In addition, depressive symptoms were measured with the eight-item Center for Epidemiological Studies Depression scale (CES-D) (18). The CES-D measures self-reported depressive symptomatology and, although not a clinical diagnostic tool, it is widely used to identify people “at risk” of depression (19). The eight-item version used in the HRS has good internal consistency (Cronbach’s α .8 in repeated measurements) and other psychometric values comparable to the full 20-item CES-D (18). A CES-D summary score was derived by summing responses to all eight dichotomous questions (“was depressed,” “everything was an effort,” “sleep was restless,” “was happy,” “felt lonely,” “enjoyed life,” “felt sad,” and “could not get going”). We dichotomized the summary score using a cut point of four or higher (≥4) to indicate elevated depressive symptoms, which is equivalent to the conventional cut point of 16 or higher on the full 20-item CES-D (18). The CES-D was not available in the first wave in 1992.

Based on a priori decision, the number of chronic conditions at each study wave was summed and categorized as 0, 1, 2, and 3 or more, which was also supported by the variable distribution. The analyses were conducted by number of diseases and by individual diseases. The disease variables were treated as time-varying variables and their value was taken from the same time point as physical functioning.

### Measurement of Physical Functioning

Physical functioning relevant for daily activities was asked about in each wave using standardized instruments (20,21). Difficulties in mobility, arm functions, and fine-motor function were self-assessed on 10 tasks, including walking one block, sitting for about 2 hours, getting up from a chair after sitting for long periods, climbing several flights of stairs without resting, climbing one flight of stairs without resting, stooping, kneeling, or crouching, reaching or extending arms above shoulder level, pulling or pushing large objects (such as a living room chair), lifting or carrying weights over 5 kilos (such as a heavy bag of groceries), and picking up a small coin from a table. Participants who reported that they had difficulty or were unable to perform the task were coded as having difficulty with the task (yes/no). These 10 items were summed to obtain a continuous physical functioning score, with higher scores indicating more severe limitations (range 0–10) (20). This composite measure has been used in previous large scale studies (21–23) and the advantage is that it allowed us to assess a broad range of physical functioning simultaneously (24–26).

### Covariates

Three time-invariant variables were included in our regression models: gender, race, and education. Race was categorized into three groups (white/Caucasian, black/African American, and Other). Education was categorized in three levels (low = less than high school, medium = high school or some college, and high = college and above). Nonhousing financial wealth was used as time-variant and was divided into tertiles (low = less than $1,000, middle = $1,000–40,000, and high = more than $40,000).

### Statistical Analysis

Study population characteristics are reported across study waves by age as proportions for categorical variables. Age-related physical functioning trajectories were assessed using linear regression analyses with generalized estimation equations (GEE) using an exchangeable correlation structure to control for the intraindividual correlation between repeated measurements (27,28). In these models, the data are structured so that measurement times (observations) are nested within participants. On average, participants provided data at 6 of the possible 10 study phases, contributing to physical functioning calculations according to their comorbidity status at each phase. The associations between disease status and physical functioning are analyzed cross-sectionally.

To examine whether the age-related trajectories were dependent on disease status, we tested disease status × age interaction terms. Age, divided into 10-year categories, represents the time variable in the model and was determined by the respondent’s age at each interview phase. In the analyses, we examined the differences in number of physical functioning difficulties and their trajectories at different ages. The analyses were conducted by number of diseases and by individual diseases. We also examined these differences in different disease combinations and according to sociodemographic characteristics. Adjusted mean estimates were calculated to represent the average level of physical functioning at different age groups and sociodemographic factors. All models were adjusted for sociodemographic factors (sex, race, education, nonhousing financial wealth, and birth cohort).

Our sensitivity analysis addressed the possibility that including those who dropped out from the study or died during the follow-up may have confounded the results because of the selection due to poor health. We repeated the main analysis by taking into account only those who remained in the study over at least six visits. The SAS 9.3 Statistical Package was used for all analyses (SAS Institute Inc., Cary, NC).

## Results

**Figure 1.**
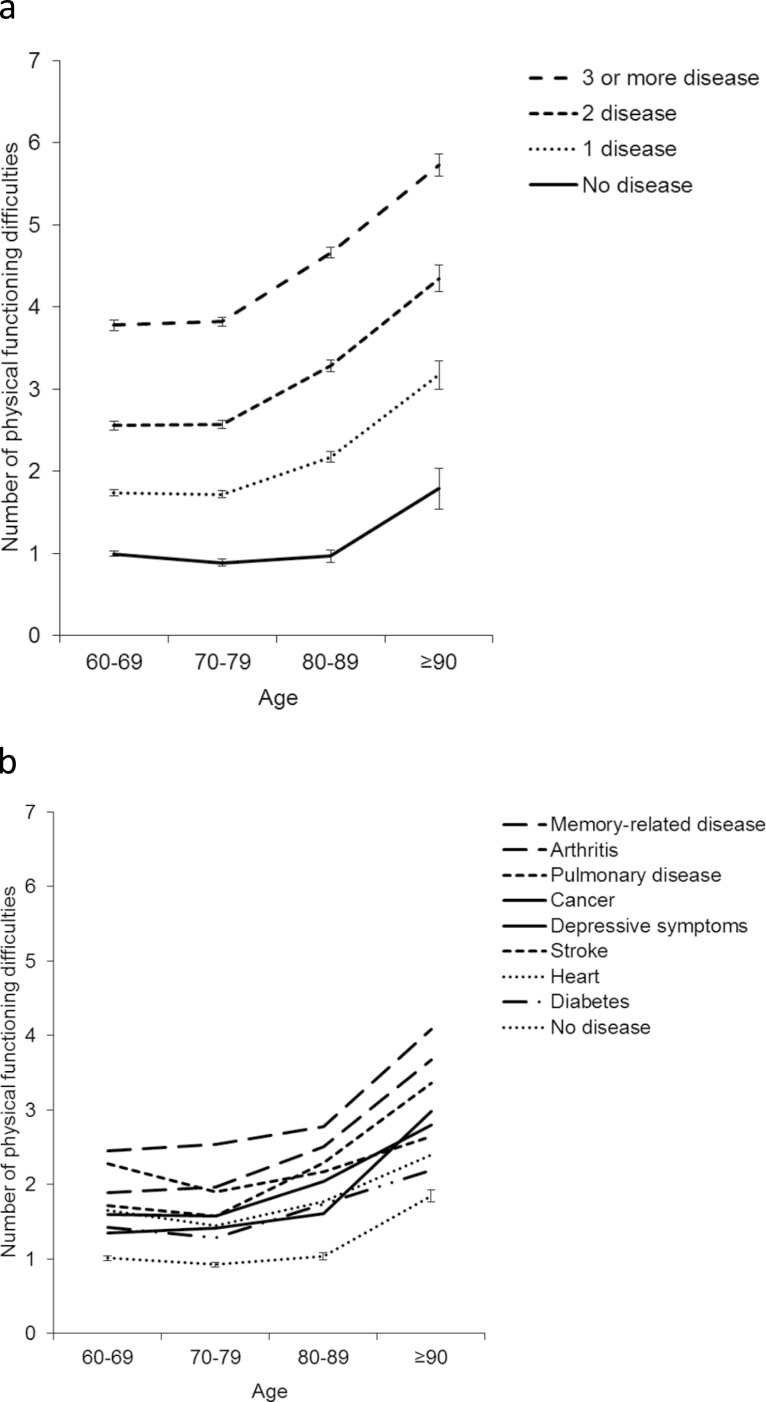
Number of physical functioning difficulties according to age and disease status. Adjusted for age and sex. (**a**) Number of diseases, (**b**) individual diseases.

The average age of the participants entering to the study was 73.0 (SD 8.1) years and they were followed biennially on average 10.2 years (median: 10 years). Table 1 shows characteristics of study population by age group. The majority of the participants were white, women and they had completed high school education or higher. The cumulative prevalence of chronic diseases increased with advancing age with arthritis, depressive symptoms, and heart disease being the most commonly reported chronic conditions. Figure 1a shows the predicted number of physical functioning difficulties according to age and disease status from the generalized estimation equation model. Age modified the relationship between number of chronic diseases and physical functioning with older participants having more physical functioning difficulties with increasing number of diseases (age × disease interaction, *p* < .0001). For example, on average 70- to 79-year-old participants with no diseases had 0.89 (95% CI: 0.85–0.93) physical functioning difficulties, with one disease it rose to 1.72 (95% CI: 1.68–1.76) difficulties, with two diseases 2.57 (95% CI: 2.52–2.62) difficulties and with three or more diseases 3.82 (95% CI: 3.76–3.88) difficulties.

**Table 1.** Characteristics of Study Population by Age Groups

	60–69	70–79	80–89	≥90
No. of observations	55,195	43,826	23,340	4,700
Men (%)	45.7	44.1	38.2	28.9
Birth cohort (%)				
<1920	0	9.2	55.8	97.0
1920–1929	5.6	47.3	42.7	3.0
1930–1939	60.6	42.1	1.5	0
≥1940	34.0	1.4	0	0
Race (%)				
White	80.4	84.1	85.7	84.8
Black	15.6	12.9	11.9	13.3
Other	4.0	3.1	2.4	1.9
Education (%)				
Less than high school	29.2	33.5	40.0	48.9
High school	51.9	50.1	46.8	39.6
College and above	19.0	16.4	13.2	11.5
Nonhousing financial wealth (%)				
Lowest tertile	33.8	30.0	28.8	34.2
Middle tertile	32.9	32.7	32.4	31.3
Highest tertile	33.3	37.4	38.8	34.5
Chronic conditions (%)				
Heart disease	18.8	29.2	38.0	41.7
Stroke	5.7	10.2	16.6	20.9
Pulmonary disease	8.6	10.7	10.6	8.2
Cancer	10.4	16.3	19.4	17.7
Diabetes	17.7	19.9	17.1	11.2
Arthritis	54.2	58.4	62.9	67.8
Depressive symptoms	29.5	28.0	33.1	40.6
Memory-related disease	2.2	4.2	10.4	19.3
Number of chronic conditions (%)				
0	23.9	16.7	11.7	9.9
1	32.4	29.5	24.7	20.4
2	21.7	25.0	26.4	26.5
≥3	22.1	28.8	37.2	43.2

*Note:* Health and Retirement Study (*n* = 127,061 person observations).

Of the individual diseases memory-related disease was associated with highest number of physical functioning difficulties across age groups. Among the participants aged 60–69 years stroke was also related to higher number of physical functioning difficulties. Among the participants aged 70 years and older arthritis and pulmonary disease were associated with significantly higher physical functioning difficulties compared with other diseases (Figure 1b). Table 2 presents the proportion of participants with no comorbid disease and with different disease combination. Arthritis was the disease that most commonly appeared alone and heart disease and depressive symptoms were most often accompanied by other diseases (with arthritis being the most comorbid disease). In Table 3, the average number of physical functioning difficulties according to specific diseases and disease combinations combining data across all age groups are shown. Memory-related disease, stroke, and pulmonary disease and arthritis, whether present alone or in combination with other diseases, were associated with the highest number of physical functioning difficulties. Participants with both memory-related disease and pulmonary disease (5.94, 95% CI: 5.55–6.34) and memory-related disease and stroke (5.75, 95% CI: 5.39–6.12) had on average the highest number of physical functioning difficulties compared with of any two concomitant chronic disease (2.90, 95% CI: 2.87–2.93).

**Table 2.** The Proportion of Observations From Participants by the Disease Status

	No. of Observations	Nothing Else	+ Any of the Following							
			Heart Disease	Stroke	Pulmonary Disease	Cancer	Diabetes	Arthritis	Depressive Symptoms	Memory-Related Disease
No disease	24,549									
Heart disease	33,975	13.3		18.0	16.3	17.5	26.2	67.2	37.9	8.1
Stroke	12,475	8.5	49.0		15.2	17.2	27.5	66.6	43.0	16.2
Pulmonary disease	12,318	9.2	44.8	15.4		20.0	21.9	71.6	45.6	8.3
Cancer	18,233	15.6	32.7	11.8	13.5		20.2	63.9	32.3	5.9
Diabetes	23,022	13.9	38.6	14.9	11.7	16.0		66.1	40.0	6.3
Arthritis	73,383	29.4	31.1	11.3	12.0	15.9	20.8		36.3	5.9
Depressive symptoms	35,921	12.9	33.8	13.8	14.7	15.8	24.3	70.7		7.5
Memory-related disease	5,040	5.9	44.1	33.2	16.6	17.9	24.1	72.6	51.7	

**Table 3.** Average Number of Physical Functioning Difficulties According to Different Disease and Disease Combinations*

	+ Any of the Following							
	Nothing Else	Heart Disease	Stroke	Pulmonary Disease	Cancer	Diabetes	Arthritis	Depressive Symptoms
	Mean (95% CI)	Mean (95% CI)	Mean (95% CI)	Mean (95% CI)	Mean (95% CI)	Mean (95% CI)	Mean (95% CI)	Mean (95% CI)
Nothing	**0.94** (0.91–0.97)							
Heart	**1.84** (1.70–1.98)							
Stroke	**2.51** (2.21–2.81)	**4.85** (4.59–5.11)						
Pulmonary disease	**2.31** (2.06–2.55)	**4.96** (4.82–5.11)	**5.47** (5.18–5.75)					
Cancer	**1.59** (1.39–1.79)	**4.22** (4.08–4.37)	**4.85** (4.57–5.13)	**4.64** (4.41–4.87)				
Diabetes	**1.45** (1.32–1.59)	**4.23** (4.11–4.36)	**4.67** (4.52–4.81)	**4.84** (4.69–4.99)	**3.71** (3.56–3.85)			
Arthritis	**2.31** (2.23–2.38)	**4.10** (4.02–4.17)	**4.62** (4.53–4.71)	**4.59** (4.50–4.68)	**3.70** (3.61–3.78)	**5.24** (4.90–5.58)		
Depressive symptoms	**1.68** (1.56–1.79)	**4.46** (4.35–4.57)	**4.88** (4.75–5.00)	**5.01** (4.89–5.14)	**4.00** (3.87–4.12)	**4.04** (3.93–4.16)	**4.34** (4.24–4.43)	
Memory-related disease	**3.23** (2.76–3.70)	**5.37** (5.01–5.72)	**5.75** (5.39–6.12)	**5.94** (5.55–6.34)	**5.30** (4.91–5.70)	**4.33** (4.21–4.46)	**5.24** (4.90–5.58)	**5.31** (4.95–5.66)
Any one disease	**1.87** (1.84–1.89)							
Any two diseases	**2.90** (2.87–2.93)							
Any three or more diseases	**4.32** (4.29–4.35)							

*Notes:* CI = confidence interval.

Bold values indicate adjusted mean estimates based on generalized estimation equations.

*Adjusted for age, sex, race, education, nonhousing financial wealth, and study cohort.

Stratified analysis by sex, race, education, and nonhousing financial wealth are shown in Supplementary Table 1. Women with any disease showed much higher levels of physical functioning difficulties than men across age groups. In addition, those with low levels of education and nonhousing financial wealth and any disease had more physical functioning difficulties compared with highest level of education and wealth, respectively. Participants with other ethnic background had lower levels of physical functioning difficulties compared with white and black participants.

A sensitivity analysis was conducted by including only those who remained in the study at least six visits and with no missing data on diseases (*n* = 83,787 person observations) replicating the main findings (Supplementary Table 2). Finally, because the 10-item physical functioning outcome includes different aspects of physical functioning, we wanted to examine whether the results would be different with less heterogeneous outcome and the analysis were repeated focusing only to the mobility items. The results were very similar for the number of diseases and the individual diseases were almost in the same order as in the 10-item physical functioning outcome. The role of memory-related diseases was highlighted in both outcomes, but arthritis and pulmonary diseases did not stand out as much with mobility outcome and with overall 10-item physical functioning outcome.

## Discussion

Using data from the U.S. HRS, an ongoing cohort study with an average of 10-year follow-up, we found that the number of chronic diseases was associated with greater burden of physical functioning difficulties in all ages. Remaining free of disease was associated with little change in physical functioning difficulties during the entire follow-up period, except after the age of 80 years. The results of this study also confirm previous findings about better physical functioning among men, well-educated, and those reporting good financial circumstances (21,29,30).

Although the association between the number of chronic diseases and physical functioning has already been reported two decades ago (14), the HRS data allowed us to examine the developmental trajectories of physical functioning using within-participant repeat data. Most of the previous comorbidity studies take into account only prevalent diseases at baseline, with a few exceptions (8). However, in this study, we treated chronic diseases as time varying variables so that newly occurring chronic diseases were also included in the analysis. This allowed us to examine the role of specific diseases and comorbidity on physical functioning in different age groups. Despite the fact that in general the number of diseases increases and functional reserve decreases with age, we observed that age modified the relationship between diseases and physical functioning with older participants having a greater level of difficulties with increasing number of chronic diseases. This is probably due to increasing disease-induced impairments and deteriorating compensatory mechanisms with aging.

For the individual diseases, we found that memory-related diseases, stroke, pulmonary diseases, and arthritis were associated with significantly higher physical functioning difficulties compared with other diseases. The strong association between memory-related diseases and physical functioning difficulties was expected because many studies have identified an independent relationship of cognitive and motor performance (10,31,32). Previous studies comparing multiple diseases have also found that stroke is among the most disabling of conditions (8,9,13,14) affecting multiple areas of functioning. Worldwide stroke is the third leading cause of the burden disease (33). Stroke-related disability often catastrophically appears after an acute event (34), which partly explains high number of physical functioning difficulties in middle-aged stroke participants. In addition, pulmonary diseases (8,14) and arthritis (35) have been shown to be strong predictors of functional disability. Functional limitations and disabilities related to pulmonary disease and arthritis often appear slowly and they impair ambulatory activities. Pulmonary diseases, especially chronic obstructive pulmonary disease, and arthritis were both among the leading causes of years lived with disability based on the Global Burden of Disease Study (36).

The results of this study also suggest that combination of different diseases may have a different impact on physical functioning. We found that memory-related disease in combination with pulmonary disease or stroke was associated with double the number of physical functioning difficulties than any two concomitant chronic diseases or these conditions alone. All these three conditions were also individually associated with high number of physical functioning difficulties, but in combination the functional consequences were amplified. Further research about these comorbid conditions, their additive roles, and functional consequences are needed.

Like with many health problems in later life, the causes of functional decline are multifactorial. In addition to chronic diseases, impairments such as loss of muscle mass and strength (sarcopenia) or cognitive decline are independently associated with functional loss (37,38). These impairments are physiological changes seen in the aging body. However, many chronic diseases can contribute to the development of these impairments, as suggested in the disablement process model (39). For example, arthritis may lead to decreased muscle strength if the person has to limit physical and everyday activities due to pain. The results of this study also support the healthy aging paradigm, ie aging is not inevitable related with diseases and associated functional decline and people are who are able to live disease free may also be able to escape functional limitations until very old age. Those participants in their 80s without any disease had more than half less functional difficulties compared with 60-year-old participants with two diseases and quarter of the limitations compared with 60-year-olds with three or more diseases.

The main strengths of the study include the prospective longitudinal design with biennially updated information on physical functioning averaging 10 years, and in some cases even 18 years, of follow-up. The results can be generalized to the U.S. adult population due to the nationally representative sample.

The limitations of the study also need to be acknowledged. First, chronic diseases were assessed by self-report and information on disease severity was not available. It is possible that self-reports may lead to under-reporting of diseases—persons who are infrequent users of health care and/or who display mild symptoms are least likely to report a doctor’s diagnosis. However, the reliability of self-reported heart disease in the HRS is shown to be highly consistent with self-reports of heart disease based on the National Health Interview Survey (National Center for Health Statistics, 1999). Second, memory-related diseases and depressive symptoms were not measured in each study wave, thus likely underestimating the role of these diseases. Third, the effect of prevalent and incident diseases on physical functioning was not differentiated in the analyses, which has potential to bias the results for diseases occurring unexpectedly and having catastrophic and rapid effects on physical functioning, such as stroke and myocardial infarction. However, based on the additional analysis, we observed that in the HRS data the number of physical functioning difficulties were almost equally associated with both prevalent and incident diseases across the age groups, and thus the bias is in unlikely state. In addition, in our analyses chronic diseases are treated as time varying variables so that newly occurring diseases were also included in the analysis. Fourth, limited list of diseases was assessed in the HRS, and we lacked information on fractures, osteoporosis, peripheral arterial disease, and Parkinson’s diseases, all of which have been shown to be important predictors of physical disability (8,9,40).

Finally, difficulties in physical functioning were also based on self-reported information. Replication using objective physical performance measures would alleviate concerns regarding potential self-reported bias. On the other hand, self-reports provide valuable information about the person’s own perception of his/her functioning in the living environment (2). The outcome measure used was a summary score of number of the physical functioning difficulties. Many previous studies have focused on single mobility items, such as walking a quarter of a mile and climbing stairs (14), which are shown to predict more severe functional problems and institutionalization (41). However, it is common that older person can have difficulties in multiple areas of physical functioning, which we sought to capture with the composite measure used in this study (24–26).

To conclude, data from a population-based sample of US adults indicate that comorbidity is associated with the number of physical functioning difficulties and age modified this relationship. Early prevention of chronic diseases and proper treatment of existing diseases are important in order to help older people to maintain good physical functioning into old age.

## Supplementary Material

Supplementary material can be found at: http://biomedgerontology.oxfordjournals.org/

## Funding

This work was supported by the EU’s Era-Age 2 program (Academy of Finland [264944] and the Swedish Research Council for Health, Working Life and Welfare [Forte, 2012-1661]). S.S. is also supported by the Academy of Finland (273850). M.K. is also supported by the UK Medical Research Council (K013351), the National Heart, Lung and Blood Institute (HL36310), the National Institute of Aging (AG034454), and a professorial fellowship from the Economic and Social Research Council. J.H. is supported by the Economic and Social Research Council (ES/K01336X/1). The funders had no role in study design, data collection and analysis, decision to publish, or preparation of the manuscript.

## Conflict of Interest

The authors declare no conflict of interest.

## Supplementary Material

Supplementary Data
