# Supplementary material for: Comorbidity and Functional Trajectories From Midlife to Old Age: The Health and Retirement Study
Source: J Gerontol A Biol Sci Med Sci. 2014 Jul 24;70(3):330–6. doi: 10.1093/gerona/glu113 (PMC4336333; doi:10.1093/gerona/glu113)
Supplement: Supplementary Data [file supp_70_3_330__index.html]

Comorbidity and Functional Trajectories From Midlife to Old Age: The Health and Retirement Study — Comorbidity and Functional Trajectories From Midlife to Old Age: The Health and Retirement Study — Supplementary Data 

# Comorbidity and Functional Trajectories From Midlife to Old Age: The Health and Retirement Study

## Supplementary Data

Data files

**Files in this Data Supplement:**

- Supplementary Data - Supplementary Data
