# Supplementary material for: Comorbidity and Functional Trajectories From Midlife to Old Age: The Health and Retirement Study
Source: J Gerontol A Biol Sci Med Sci. 2014 Jul 24;70(3):330–6. doi: 10.1093/gerona/glu113 (PMC4336333; doi:10.1093/gerona/glu113)
Supplement: Supplementary Data [file supp_glu113_SupplementTables_revised.doc]

**Supplement Table 1**. Average number of physical functioning difficulties according number of diseases, sex, race, education and non-housing financial wealth*.

|  | Number of diseases | | | | | | | | | | | |
| --- | --- | --- | --- | --- | --- | --- | --- | --- | --- | --- | --- | --- |
|  | 0 | | | 1 | | | 2 | | | 3 or more | | |
|  | Mean | 95% CI | | Mean | 95% CI | | Mean | 95% CI | | Mean | 95% CI | |
| **Overall** |  |  |  |  |  |  |  |  |  |  |  |  |
| 60-69 | 1.05 | 0.98 | 1.12 | 1.76 | 1.69 | 1.83 | 2.54 | 2.46 | 2.61 | 3.70 | 3.62 | 3.79 |
| 70-79 | 1.03 | 0.96 | 1.11 | 1.82 | 1.75 | 1.89 | 2.63 | 2.55 | 2.70 | 3.81 | 3.73 | 3.89 |
| 80-89 | 1.26 | 1.16 | 1.36 | 2.42 | 2.33 | 2.51 | 3.48 | 3.39 | 3.57 | 4.80 | 4.71 | 4.89 |
| ≥90 | 2.15 | 1.89 | 2.41 | 3.51 | 3.32 | 3.70 | 4.65 | 4.48 | 4.83 | 5.97 | 5.82 | 6.12 |
|  |  |  |  |  |  |  |  |  |  |  |  |  |
| **Sex** |  |  |  |  |  |  |  |  |  |  |  |  |
| Men |  |  |  |  |  |  |  |  |  |  |  |  |
| 60-69 | 0.84 | 0.75 | 0.94 | 1.46 | 1.36 | 1.55 | 2.09 | 1.98 | 2.21 | 3.28 | 3.15 | 3.41 |
| 70-79 | 0.82 | 0.72 | 0.93 | 1.47 | 1.37 | 1.57 | 2.22 | 2.11 | 2.32 | 3.36 | 3.24 | 3.48 |
| 80-89 | 1.00 | 0.86 | 1.14 | 2.00 | 1.86 | 2.13 | 2.98 | 2.84 | 3.12 | 4.32 | 4.18 | 4.46 |
| ≥90 | 2.00 | 1.60 | 2.39 | 3.04 | 2.75 | 3.32 | 4.02 | 3.73 | 4.31 | 5.68 | 5.41 | 5.95 |
| Women |  |  |  |  |  |  |  |  |  |  |  |  |
| 60-69 |  |  |  |  |  |  |  |  |  |  |  |  |
| 70-79 | 1.23 | 1.14 | 1.33 | 2.04 | 1.95 | 2.14 | 2.94 | 2.84 | 3.05 | 4.09 | 3.97 | 4.20 |
| 80-89 | 1.25 | 1.14 | 1.35 | 2.15 | 2.05 | 2.25 | 3.01 | 2.90 | 3.11 | 4.22 | 4.11 | 4.33 |
| ≥90 | 1.54 | 1.40 | 1.67 | 2.80 | 2.68 | 2.92 | 3.92 | 3.79 | 4.04 | 5.22 | 5.10 | 5.34 |
|  | Number of diseases | | | | | | | | | | | |
|  | 0 | | | 1 | | | 2 | | | 3 or more | | |
|  | Mean | 95% CI | | Mean | 95% CI | | Mean | 95% CI | | Mean | 95% CI | |
| **Race** |  |  |  |  |  |  |  |  |  |  |  |  |
| White |  |  |  |  |  |  |  |  |  |  |  |  |
| 60-69 | 0.97 | 0.91 | 1.03 | 1.65 | 1.59 | 1.72 | 2.36 | 2.29 | 2.43 | 3.50 | 3.42 | 3.58 |
| 70-79 | 0.93 | 0.87 | 1.00 | 1.71 | 1.65 | 1.77 | 2.50 | 2.43 | 2.57 | 3.66 | 3.58 | 3.74 |
| 80-89 | 1.12 | 1.02 | 1.22 | 2.28 | 2.19 | 2.36 | 3.35 | 3.26 | 3.44 | 4.66 | 4.57 | 4.74 |
| ≥90 | 2.05 | 1.78 | 2.32 | 3.44 | 3.24 | 3.64 | 4.47 | 4.29 | 4.66 | 5.90 | 5.75 | 6.06 |
| Black |  |  |  |  |  |  |  |  |  |  |  |  |
| 60-69 | 0.82 | 0.66 | 0.98 | 1.61 | 1.45 | 1.77 | 2.67 | 2.48 | 2.85 | 3.87 | 3.68 | 4.06 |
| 70-79 | 0.82 | 0.63 | 1.01 | 1.71 | 1.54 | 1.88 | 2.59 | 2.40 | 2.79 | 3.94 | 3.75 | 4.14 |
| 80-89 | 1.30 | 1.02 | 1.58 | 2.52 | 2.25 | 2.79 | 3.51 | 3.25 | 3.77 | 4.94 | 4.69 | 5.19 |
| ≥90 | 2.18 | 1.44 | 2.92 | 3.27 | 2.76 | 3.78 | 5.10 | 4.64 | 5.57 | 5.70 | 5.33 | 6.07 |
| Other |  |  |  |  |  |  |  |  |  |  |  |  |
| 60-69 |  |  |  |  |  |  |  |  |  |  |  |  |
| 70-79 | 0.83 | 0.56 | 1.09 | 1.75 | 1.49 | 2.01 | 2.86 | 2.52 | 3.20 | 4.11 | 3.76 | 4.46 |
| 80-89 | 0.91 | 0.55 | 1.27 | 1.75 | 1.47 | 2.03 | 2.64 | 2.31 | 2.96 | 3.65 | 3.29 | 4.00 |
| ≥90 | 1.39 | 0.69 | 2.10 | 2.42 | 2.01 | 2.84 | 3.70 | 3.08 | 4.32 | 4.52 | 4.04 | 5.00 |
|  |  |  |  |  |  |  |  |  |  |  |  |  |

|  | Number of diseases | | | | | | | | | | | |
| --- | --- | --- | --- | --- | --- | --- | --- | --- | --- | --- | --- | --- |
|  | 0 | | | 1 | | | 2 | | | 3 or more | | |
|  | Mean | 95% CI | | Mean | 95% CI | | Mean | 95% CI | | Mean | 95% CI | |
| **Education** |  |  |  |  |  |  |  |  |  |  |  |  |
| Less than high school | |  |  |  |  |  |  |  |  |  |  |  |
| 60-69 | 1.35 | 1.20 | 1.50 | 2.17 | 2.03 | 2.32 | 3.12 | 2.96 | 3.28 | 4.33 | 4.17 | 4.48 |
| 70-79 | 1.24 | 1.09 | 1.39 | 2.14 | 1.99 | 2.28 | 3.09 | 2.94 | 3.25 | 4.41 | 4.26 | 4.56 |
| 80-89 | 1.56 | 1.37 | 1.74 | 2.73 | 2.56 | 2.91 | 3.92 | 3.74 | 4.10 | 5.31 | 5.14 | 5.48 |
| ≥90 | 2.70 | 2.30 | 3.11 | 3.66 | 3.36 | 3.97 | 5.07 | 4.79 | 5.36 | 6.40 | 6.17 | 6.64 |
| High school |  |  |  |  |  |  |  |  |  |  |  |  |
| 60-69 | 1.09 | 0.98 | 1.20 | 1.84 | 1.73 | 1.95 | 2.58 | 2.46 | 2.70 | 3.68 | 3.55 | 3.81 |
| 70-79 | 1.12 | 1.00 | 1.23 | 1.94 | 1.83 | 2.05 | 2.68 | 2.56 | 2.80 | 3.83 | 3.70 | 3.95 |
| 80-89 | 1.36 | 1.20 | 1.51 | 2.56 | 2.42 | 2.69 | 3.55 | 3.41 | 3.69 | 4.88 | 4.73 | 5.02 |
| ≥90 | 1.96 | 1.61 | 2.31 | 3.73 | 3.46 | 4.01 | 4.65 | 4.39 | 4.90 | 6.07 | 5.84 | 6.30 |
| College and above | |  |  |  |  |  |  |  |  |  |  |  |
| 60-69 | 0.92 | 0.79 | 1.05 | 1.36 | 1.23 | 1.50 | 1.92 | 1.77 | 2.08 | 2.96 | 2.75 | 3.16 |
| 70-79 | 0.95 | 0.81 | 1.09 | 1.48 | 1.34 | 1.61 | 2.21 | 2.05 | 2.36 | 3.15 | 2.96 | 3.33 |
| 80-89 | 1.07 | 0.87 | 1.27 | 2.08 | 1.89 | 2.27 | 3.04 | 2.83 | 3.25 | 4.11 | 3.88 | 4.34 |
| ≥90 | 1.96 | 1.23 | 2.69 | 3.37 | 2.88 | 3.86 | 4.49 | 4.01 | 4.97 | 5.47 | 4.98 | 5.95 |
|  |  |  |  |  |  |  |  |  |  |  |  |  |

|  | Number of diseases | | | | | | | | | | | |
| --- | --- | --- | --- | --- | --- | --- | --- | --- | --- | --- | --- | --- |
|  | 0 | | | 1 | | | 2 | | | 3 or more | | |
|  | Mean | 95% CI | | Mean | 95% CI | | Mean | 95% CI | | Mean | 95% CI | |
| **Non-housing wealth** | |  |  |  |  |  |  |  |  |  |  |  |
| Lowest tertile |  |  |  |  |  |  |  |  |  |  |  |  |
| 60-69 | 1.01 | 0.90 | 1.13 | 1.88 | 1.76 | 1.99 | 2.98 | 2.85 | 3.10 | 4.24 | 4.11 | 4.36 |
| 70-79 | 0.93 | 0.80 | 1.06 | 1.86 | 1.74 | 1.98 | 2.88 | 2.74 | 3.01 | 4.29 | 4.16 | 4.41 |
| 80-89 | 1.38 | 1.19 | 1.57 | 2.54 | 2.37 | 2.72 | 3.72 | 3.54 | 3.89 | 5.30 | 5.15 | 5.46 |
| ≥90 | 2.51 | 2.01 | 3.00 | 3.26 | 2.90 | 3.62 | 4.80 | 4.48 | 5.13 | 6.19 | 5.95 | 6.43 |
| Middle tertile |  |  |  |  |  |  |  |  |  |  |  |  |
| 60-69 | 0.83 | 0.72 | 0.93 | 1.58 | 1.47 | 1.68 | 2.35 | 2.23 | 2.47 | 3.58 | 3.44 | 3.71 |
| 70-79 | 0.77 | 0.66 | 0.88 | 1.65 | 1.54 | 1.76 | 2.43 | 2.31 | 2.55 | 3.68 | 3.55 | 3.81 |
| 80-89 | 1.03 | 0.88 | 1.17 | 2.17 | 2.03 | 2.32 | 3.30 | 3.15 | 3.45 | 4.47 | 4.33 | 4.61 |
| ≥90 | 2.03 | 1.62 | 2.44 | 3.50 | 3.18 | 3.82 | 4.53 | 4.23 | 4.84 | 5.55 | 5.29 | 5.81 |
| Highest tertile |  |  |  |  |  |  |  |  |  |  |  |  |
| 60-69 | 0.85 | 0.74 | 0.97 | 1.50 | 1.38 | 1.61 | 2.08 | 1.95 | 2.21 | 3.07 | 2.92 | 3.23 |
| 70-79 | 0.86 | 0.74 | 0.98 | 1.53 | 1.41 | 1.65 | 2.25 | 2.12 | 2.37 | 3.26 | 3.12 | 3.40 |
| 80-89 | 1.04 | 0.88 | 1.20 | 2.15 | 2.01 | 2.29 | 3.01 | 2.86 | 3.16 | 4.18 | 4.03 | 4.33 |
| ≥90 | 1.72 | 1.35 | 2.09 | 3.16 | 2.87 | 3.44 | 4.12 | 3.85 | 4.38 | 5.52 | 5.26 | 5.77 |

Notes: *Adjusted for age, sex, race, education, non-housing financial wealth and birth cohort. Interactions sex*age*disease status (p= 0.26), race*age*disease status (p= 0.005), education*age*disease status (p= 0.40), non-housing financial wealth*age*disease status (p= 0.02).

**Supplement Table 2**. Average number of physical functioning difficulties according number of diseases among participants with six or more study visits and no missing data on diseases* (n=83,787 person observations).

|  | Number of diseases | | | | | | | | | | | |
| --- | --- | --- | --- | --- | --- | --- | --- | --- | --- | --- | --- | --- |
|  | 0 | | | 1 | | | 2 | | | 3 or more | | |
|  | Mean | 95% CI | | Mean | 95% CI | | Mean | 95% CI | | Mean | 95% CI | |
| 60-69 | 1.05 | 0.93 | 1.17 | 1.68 | 1.56 | 1.80 | 2.45 | 2.32 | 2.57 | 3.52 | 3.39 | 3.66 |
| 70-79 | 1.19 | 1.06 | 1.31 | 1.89 | 1.77 | 2.02 | 2.62 | 2.50 | 2.75 | 3.73 | 3.60 | 3.86 |
| 80-89 | 1.39 | 1.23 | 1.56 | 2.38 | 2.24 | 2.51 | 3.31 | 3.17 | 3.45 | 4.46 | 4.32 | 4.60 |
| ≥90 | 2.15 | 1.77 | 2.54 | 3.40 | 3.14 | 3.67 | 4.28 | 4.04 | 4.52 | 5.48 | 5.29 | 5.68 |

Notes: * Adjusted for age, sex, race, education, non-housing financial wealth and birth cohort.
